# Comparative Study of the Antimicrobial Activity of Selenium Nanoparticles With Different Surface Chemistry and Structure

**DOI:** 10.3389/fbioe.2020.624621

**Published:** 2021-01-25

**Authors:** Nenad Filipović, Dušan Ušjak, Marina T. Milenković, Kai Zheng, Liliana Liverani, Aldo R. Boccaccini, Magdalena M. Stevanović

**Affiliations:** ^1^Institute of Technical Sciences of the Serbian Academy of Sciences and Arts, Belgrade, Serbia; ^2^Department of Microbiology and Immunology, University of Belgrade-Faculty of Pharmacy, Belgrade, Serbia; ^3^Department of Materials Science and Engineering, Institute of Biomaterials, University of Erlangen-Nuremberg, Erlangen, Germany

**Keywords:** selenium nanoparticles (SeNPs), nanocomposites, antimicrobial activity, inhibition of biofilms, dual-species biofilm

## Abstract

Although selenium nanoparticles (SeNPs) have gained attention in the scientific community mostly through investigation of their anticancer activity, a great potential of this nanomaterial was recognized recently regarding its antimicrobial activity. The particle form, size, and surface chemistry have been recognized as crucial parameters determining the interaction of nanomaterials with biological entities. Furthermore, considering a narrow boundary between beneficial and toxic effects for selenium per se, it is clear that investigations of biomedical applications of SeNPs are very demanding and must be done with great precautions. The goal of this work is to evaluate the effects of SeNPs surface chemistry and structure on antimicrobial activity against several common bacterial strains, including *Staphylococcus aureus* (ATCC 6538), *Enterococcus faecalis* (ATCC 29212), *Bacillus subtilis* (ATCC 6633), and *Kocuria rhizophila* (ATCC 9341), as well as *Escherichia coli* (ATCC 8739), *Salmonella* Abony (NCTC 6017), *Klebsiella pneumoniae* (NCIMB 9111) and *Pseudomonas aeruginosa* (ATCC 9027), and the standard yeast strain *Candida albicans* (ATCC 10231). Three types of SeNPs were synthesized by chemical reduction approach using different stabilizers and reducing agents: (i) bovine serum albumin (BSA) + ascorbic acid, (ii) chitosan + ascorbic acid, and (iii) with glucose. A thorough physicochemical characterization of the obtained SeNPs was performed to determine the effects of varying synthesis parameters on their morphology, size, structure, and surface chemistry. All SeNPs were amorphous, with spherical morphology and size in the range 70–300 nm. However, the SeNPs obtained under different synthesis conditions, i.e. by using different stabilizers as well as reducing agents, exhibited different antimicrobial activity as well as cytotoxicity which are crucial for their applications. In this paper, the antimicrobial screening of the selected systems is presented, which was determined by the broth microdilution method, and inhibitory influence on the production of monomicrobial and dual-species biofilm was evaluated. The potential mechanism of action of different systems is proposed. Additionally, the cytotoxicity of SeNPs was examined on the MRC-5 cell line, in the same concentration interval as for antimicrobial testing. It was shown that formulation SeNPs-BSA expressed a significantly lower cytotoxic effect than the other two formulations.

## Introduction

The emergence of nanotechnology on the scientific scene greatly expanded the application potential for numerous materials. Thanks to the fact that materials with nanometer dimensions exhibit properties different from bulk material, some of them have found a completely new application field. Elemental selenium is one of those materials. Nowadays, selenium is well-known as an essential micronutrient of fundamental importance to human and animal health (Rayman, 2000). As the 21st amino acid, seleno-cysteine is a constituent of many selenoproteins including glutathione peroxidases (GPx) and thioredoxin reductase (TrxR). These enzymes are of great significance in antioxidant processes. In addition, selenium is recognized as a good candidate for cancer therapy and prevention, as an anti-inflammatory agent, for treatment of cardiovascular diseases and thyroidal disorders, and to be actively engaged in bone and muscle metabolism (Clark, 1996; Ip, 1998; Rayman, 2000; Moreno-Reyes et al., 2001; Zeng and Combs, 2008; Fernandes and Gandin, 2015). It is necessary to emphasize that the biological activity of Se depends on its chemical form and structure. For example, elemental selenium is insoluble and for a long time had been considered biologically inert. Consequently, in the past, dominant selenium forms used in biomedical investigations were selenomethionine (SeMet), selenocysteine, methylselenocysteine, and sodium selenite. Nevertheless, with nanoscience expansion, elemental selenium nanoparticles (SeNPs) emerged as a new selenium form, which offered some advantages in comparison with other selenium formulations. In several works, it was shown that SeNPs provides a reduced risk of selenium toxicity, but the same bioavailability and efficacy in increasing the activities of selenoenzimes compared with Se-Met and selenite (Zhang et al., 2001, 2008; Wang et al., 2007). These findings encourage many authors to primarily investigate the anticancer activity of SeNPs, bare or conjugated with other anticancer agents (Zhang et al., 2013; Xia et al., 2015; Maiyo and Singh, 2017; Khurana et al., 2019). One of the pioneering work that reported the antimicrobial activity of SeNPs was done by Webster and Tran (2011). In this paper, the authors demonstrated that SeNPs with a diameter of around 100 nm significantly inhibit the growth of *Staphylococcus aureus* at a concentration as low as 7.8 μg/mL. In the following years, interest in the antimicrobial activity of SeNPs increased gradually among scientists, although this selenium form was not such efficient in killing bacteria as metallic and metal oxide nanoparticles (Ag, ZnO, and CuO). This interest was supported by the facts that selenium represents an essential micronutrient with diverse beneficial effects and that nanosized selenium broadened the window of its safe usage. An especially interesting field of research regarding the antimicrobial activity of SeNPs, which recently gained attention, is the inhibition of biofilm formation and activity against resistant microbial strains (Wang and Webster, 2013; Shakibaie et al., 2015a; Guisbiers et al., 2017; Prateeksha Singh et al., 2017; Cremonini et al., 2018; Tran et al., 2019). Bacterial biofilm is considered as the ultimate level of bacteria strategy in dealing with the immune system and antibiotics. Polymicrobial biofilms even enhanced this resistance due to the microbial synergy and therefore increased the risk of serious health complications (Carolus et al., 2019). Very often, polymicrobial infections originate from a mixture of bacteria and fungi. A significant amount of these infections, including those of *C. albicans* and *Staphylococcus* species, are often associated with high mortality rates (Feng et al., 2014). *C. albicans* and *Staphylococcus* species were co-isolated from various biofilm-associated diseases, including periodontitis, cystic fibrosis, denture stomatitis, urinary tract, burn wound infections, and infections of medical devices such as central venous catheters (Adam et al., 2002; Gupta et al., 2005; Valenza et al., 2008; Cuesta et al., 2010). Various biofilm-specific mechanisms of *C. albicans* provide additional protection to *S. aureus* against antimicrobial agents and host immune responses (Kong et al., 2016; Vila et al., 2019). For example, vancomycin tolerance, mediated by β-1,3-glucans, may be increased by more than 1000-fold (Harriott and Noverr, 2009). On the other hand, *S. aureus* contributes to *C. albicans* immune resistance, as well as tolerance toward antifungal agents such as miconazole (Carolus et al., 2019). Due to the complex nature of polymicrobial infections, finding their efficient treatment is very challenging (Koo et al., 2017). According to our knowledge, the activity of SeNPs against dual-species biofilms has not been reported so far.

In this framework, the present work aims to examine the effects of surface chemistry on the antimicrobial activity of three different SeNPs formulations. Accordingly, two of them were prepared by variation of stabilizing agents (BSA or chitosan) while using the same reductant, ascorbic acid. The third type was synthesized, only by reduction with glucose. Although widely accepted as just a reducing agent, glucose might also act as a stabilizing agent (Liu et al., 2006). For instance, it is known that β-D-glucose can easily both reduce and stabilize NPs by adjusting the pH environment in an aqueous medium.

All SeNPs formulations were further used for antimicrobial testing on four Gram-positive bacteria (*S. aureus, Enterococcus faecalis, Bacillus subtilis*, and *Kocuria rhizophila*) as well on four Gram-negative bacteria (*Escherichia coli, Salmonella* Abony, *Klebsiella pneumoniae*, and *Pseudomonas aeruginosa*), and on the standard yeast strain *Candida albicans*. Based on obtained results regarding antimicrobial activity, physicochemical properties, and cytotoxicity, one formulation of SeNPs was selected for the investigation of antibiofilm activity on monomicrobial and dual-species biofilms. For this purpose, several clinical isolates of *S. aureus* and *C. albicans* were used.

## Materials and Methods

### Materials

All reagents used in synthesis and for other experimental procedures were analytical grade. Sodium selenite, BSA, and chitosan (medium molecular weight) were purchased from *Sigma Aldrich*, while ascorbic acid and glucose were obtained from *BDH Prolabo* and *Zorka Pharma*, respectively. Double distilled water was used for the preparation of solutions during the synthesis procedure.

### Synthesis of SeNPs

SeNPs were synthesized by a chemical reduction approach. The experiments were planned by DOE methodology, and parameters were optimized concerning the high SeNPs yielding percent and without compromising the particle size, morphology, and amorphous nature. Three different formulations were obtained by combining different stabilizing and reducing agents: (i) SeNPs stabilized with BSA (SeNPs-BSA); (ii) SeNPs stabilized with chitosan (SeNPs-Chit); and (iii) SeNPs synthesized only by reduction with glucose (SeNPs-Gluc). Sodium selenite was used as a precursor for all samples. The SeNPs-BSA and SeNPs-Chit were obtained by reduction with ascorbic acid. Briefly, sodium selenite was dissolved in distilled water at a concentration of 0.02 M (12.5 mL). In separate flasks, 0.125 M solutions of ascorbic acid were prepared (10 mL). These solutions were then mixed with solutions of chitosan or BSA (5 mL at concentration 0.87% w/w). In the final step, the prepared solution of sodium selenite was added dropwise. Reaction mixtures were stirred on a magnetic stirrer (1,500 rpm) until their colors change to red-orange. The mass ratio between sodium selenite and chitosan or BSA was adjusted to 1:1. For the third formulation (SeNPs-Gluc), glucose was firstly dissolved in distilled water at a concentration of 0.055 M (25 mL). This solution was then placed on a magnetic stirrer, heated up to 130°C, and stirred at 1,500 rpm. The 0.017 M solution of sodium selenite (10 mL) was introduced drop by drop immediately after heating started. After ~1 h, the solution became orange/reddish, as a sign of complete reduction and conversion to Se^0^ nanoparticles. During the preparation of all samples, reaction vessels were covered with aluminum foil to prevent photo-oxidation and crystallization of obtained SeNPs. After preparation, colloidal solutions of SeNPs were stocked at 4–8°C. For FTIR and XRD measurements, samples were lyophilized in alpha 1-4 LD freeze dryer (Martin Christ) at 0.36 mbar.

### FTIR Spectroscopy

The qualitative analysis of the samples was performed by FTIR spectroscopy. The FTIR spectra were recorded on spectrometer Nicolet iS10 (Thermo Fisher Scientific, Waltham, MA, USA), using attenuated total reflectance (ATR) mode. Measurements were performed in a spectral range of 400–4,000 cm^−1^ with a resolution of 4 cm^−1^ and the number of scans was 32.

### X-Ray Diffraction (XRD)

X-ray diffraction spectra were obtained on an X-ray diffractometer, Philips PW 1,050 diffractometer with Cu-Kα radiation (Ni filter). The samples were scanned in the 2θ range of 10° to 60°, with a scanning step width of 0.05°, and 2 s per step.

### Morphology Investigation

The morphology of as-synthesized SeNPs was analyzed by FESEM (Auriga Base, Carl Zeiss). For SEM analysis, the droplets of samples were placed on aluminum tape and dried at room temperature. Dried samples were then coated with gold using a Sputter Coater (Q150T, Quorum Technologies).

### Diffracted Light Scattering and Zeta Potential

The Zeta-potential of as-synthesized SeNPs was measured using a Zetasizer Nano ZS (Malvern Instruments, UK) instrument with a 4 mW HeNe laser (633 nm) and a light scattering detector positioned at 90°. The hydrodynamic particle sizes of samples were determined by dynamic light scattering (DLS, Zetasizer Nano ZS) analysis at 25°C, setting a minimum of 10 and a maximum of 100 runs per measurement. For the measurements, the as-synthesized SeNPs were used without dilution. The analyses were performed in triplicate.

### Quantitative Determination of Se

A Thermo Scientific iCap 6500 Duo instrument was used for the determination of Se concentration in obtained samples. Working solutions of selenium were produced by appropriate dilutions of the corresponding stock solutions with 2.5% nitric acid (HNO_3_). Working standards were prepared from the multi-element standard solution, MES-21-1 (AccuStandrad, USA) in the following concentrations: 10 ppb, 20 ppb, 50 ppb, 100 ppb, 0.2 ppm, 0.5 ppm, 1 ppm, and 2 ppm.

### Antimicrobial Activity

Minimum inhibitory concentrations (MICs) of SeNPs-BSA, SeNPs-Chit, SeNPs-Gluc, and Na_2_SeO_3_ against standard microbial strains, were determined by the broth microdilution method (CLSI, 2017). Each sample was diluted with microbial growth media to final concentrations ranging from 400 to 12.5 μg/mL and transferred into 96-well polystyrene microtiter plates (Sarstedt, Germany). The wells were further supplemented with bacterial suspensions (~10^6^ colony-forming unit per mL–CFU/mL), as well as *C. albicans* suspension (~10^7^ CFU/mL), and incubated for 24 h at 35°C. Mueller-Hinton broth (MHB; HiMedia, India) and Sabouraud Dextrose Broth (SDB; Torlak, Serbia) were used as growth media for bacteria and yeast, respectively. MICs were determined as the lowest concentrations in which there was no visible growth of microorganisms.

### Morphology Observation by Optical Microscopy

Optical microscopy was used for examination of the impact of SeNPs treatment on the morphology and growth of the pathogens. For this purpose, Gram-stained smears were prepared from mixed *S. aureus* and *C. albicans* cultures in Trypton Soy Broth (Torlak, Serbia) supplemented with an additional 1% (w/v) glucose (TSBG) supplemented with SeNPs-BSA, SeNPs-Chit, or SeNPs-Gluc, at 6.4 μg/mL. In addition, control was prepared without the addition of any Se formulation. The images were taken from OPTICA B-500MET light microscope (Optica SRL, Italy). Images were collected with OPTIKAM PRO 8LT−4083.18 camera equipped with a scientific-grade CCD sensor.

### Antibiofilm Activity

The ability of SeNPs-BSA to inhibit biofilm production of *S. aureus* and *C. albicans* was evaluated by the method described by Stepanović et al. (2007). Both, type strains (*S. aureus* ATCC 6538 and *C. albicans* ATCC 10231) and clinical isolates were included in this experiment (Supplementary Table 1). TSBG was used as a growth medium for both bacteria and yeasts. According to the Clinical and Laboratory Standards Institute antimicrobial susceptibility testing standards, MHB is a preferred medium for the broth-microdilution method (CLSI, 2017). When it comes to the biofilm assay, Stepanović et al. recommend TSBG as the most suitable medium for staphylococcal biofilm cultivation (Stepanović et al., 2007). Scaffaro et al. also used this medium for the cultivation of dual-species biofilms produced by *S. aureus* and *C. albicans* (Scaffaro et al., 2018). SeNPs-BSA were added to 96-well polystyrene microtiter plates at concentrations ranging from 6.4 to 1.6 μg/mL, while TSBG supplemented with an equal concentration of BSA was used as a positive control. Previously we have tested a wider spectrum of concentrations against different microorganisms, and based on the obtained results (data not presented), it was determined that concentrations higher than 6.4 ug/ml were not more effective in the inhibition of biofilm production. The incubation of the strains (~10^6^ CFU/mL) was carried out at 35°C for 24 h. Planktonic cells were then washed off with Phosphate-Buffered Saline (PBS; pH 7.2), and the remaining surface-bound biofilm was fixed by air-drying at 60°C for 1 h and stained with 0.5% safranin (HiMedia, India) for 15 min. Finally, the stain was extracted with 96% (v/v) ethanol and its optical density (OD) was measured at 492 nm by using EZ Read 400 ELISA reader (Biochrom, USA).

### Dual-Species Biofilm Inhibitory Activity

Dual-species biofilm production of treated combinations of *S. aureus* and *C. albicans* strains was evaluated on polystyrene surface of 96-well microtiter plates, as well as on radiopaque polyurethane surface of Two-Lumen Central Venous Catheter (CVC; Arrow International, USA). In both cases, the material was firstly seeded with *C. albicans* (~10^6^ CFU/mL) and incubated for 1 h at 35°C to allow the yeast-to-hyphae transition. Afterward, *S. aureus* (~10^6^ CFU/mL) was added, putatively binding to previously formed *C. albicans* hyphae, and incubated for an additional 24 h at 35°C (Scaffaro et al., 2018). Vein-indwelling tube of CVC was cut into small pieces, which were then scrubbed with water and soap, rinsed with hot tap water and distilled water, three times each, and finally autoclaved at 121°C for 15 min. Washing, fixation, and staining procedures were performed as described above. Evaluation of biofilm production on CVC was performed using the modified biofilm index, based on digital image analysis for color brightness quantification, as described by Yamamoto et al. (2007).

In addition, the colony counting method was employed to determine the relative numbers of microorganisms in mixed biofilms. Briefly, dual-species biofilms composed of treated *S. aureus* ATCC 6538 and *C. albicans* 10231, formed as described above in polyethylene tubes, were washed three times with PBS, and the remaining biofilm cells were resuspended in 3 mL of PBS with vigorous vortexing for 1.5 min. Serial 10-fold dilutions were prepared and plated on mannitol salt agar (MSA; Torlak, Serbia) and Sabouraud dextrose agar (SDA; Torlak, Serbia) to count the colonies of *S. aureus* and *C. albicans*, respectively, following the incubation for 24 h at 37°C.

### Reduction of Dual-Species Biofilm Cell Viability

The viability of dual-species biofilm cells, following the treatment with SeNPs-BSA, was estimated by using 2,3,5-triphenyl-2H-tetrazolium chloride (TTC; Sigma-Aldrich, USA) (Sabaeifard et al., 2014). After the overnight incubation, the plates were filled with 120 μL of fresh TSBG and 30 μL of 0.5% (w/v) TTC, immediately after the washing procedure, skipping the air-drying fixation step and incubated for another 6 h at 35°C. ODs were measured at 405 nm.

### Statistical Analysis

Each test was repeated three times. Results are presented as mean values ± standard deviations (SDs). All the groups were confirmed to exhibit normal distribution by the Shapiro–Wilk test. Experimental and control groups were compared by one-way analysis of variance (ANOVA), followed by Tukey's *post hoc* test. Calculations were performed using the SPSS Statistics, IBM SPSS Software, v24.0 (IBM, USA).

### Cytotoxicity of SeNPs

MRC-5 cells were cultured in Dulbecco's modified Eagle medium supplemented with 10% FBS and 1% penicillin/streptomycin mix, at 37°C in 5% CO_2_ and high humidity. Cells were split upon reaching confluency.

MTT (3-[4,5-dimethylthiazol-2-yl]-2,5-diphenyltetrazolium bromide) assay was used to assess cytotoxicity of SeNPs. Cells were seeded at a density of 2 × 10^4^ cells/well in 96-well plates and incubated for 24 h at 37°C in 5% CO_2_ before SeNPs treatment. Then, the medium was discarded and 200 μl/well of fresh medium containing different concentrations of SeNPs samples (SeNPs-Gluc, SeNPs-Chit, SeNPs-BSA) was added to the wells. After 24 h of incubation at 37°C in 5% CO_2_, the treatment medium was replaced with fresh medium, followed by the addition of 20 μl/well of 5 mg/ml MTT solution. After 4 h of incubation, the medium was carefully discarded and formazan crystals dissolved using 200 μl/well of DMSO. Absorbance was recorded at 570 nm. Results are expressed relative to the absorbance of control cells.

## Results and Discussion

### Synthesis of SeNPs

Several different techniques have been employed in SeNPs synthesis, so far. These techniques can be roughly classified into three groups: chemical reduction methods, conversion by physical field (microwave-assisted or laser ablation), and biosynthesis approach. All of them offer some advantages and drawbacks and the majority can be found in the following review papers (Chaudhary et al., 2014, 2016; Wadhwani et al., 2016; Hosnedlova et al., 2018; Sakr et al., 2018; Shi et al., 2020). Chemical reduction methods are simple, economical, fast, and suitable for production on a larger scale. The choice of reducing and stabilizing agents as well as their concentrations are crucial from the point of particle size and morphology. In our previous work (Stevanović et al., 2015) we have already determined the optimal ratio between ascorbic acid and sodium selenite, which was also confirmed in a paper, recently published by Chung et al. (2020). BSA and chitosan are selected as models of stabilizing agents since they represent different types of macromolecules. Albumins are generally the most abundant globular proteins in serum and provide good biocompatibility while chitosan is a cationic, linear polysaccharide with documented antibacterial properties. Their application in the stabilization of SeNPs was already reported in many papers (Valueva et al., 2007; Chen et al., 2015; Lara et al., 2018; Filipović et al., 2019; Chung et al., 2020; Shi et al., 2020). The third formulation of SeNPs is designed to obtain nanoparticles without using any macromolecule as a stabilizing agent while not compromising parameters such as crystallinity, size, and morphology.

### FTIR Characterization

**Figure 1:**
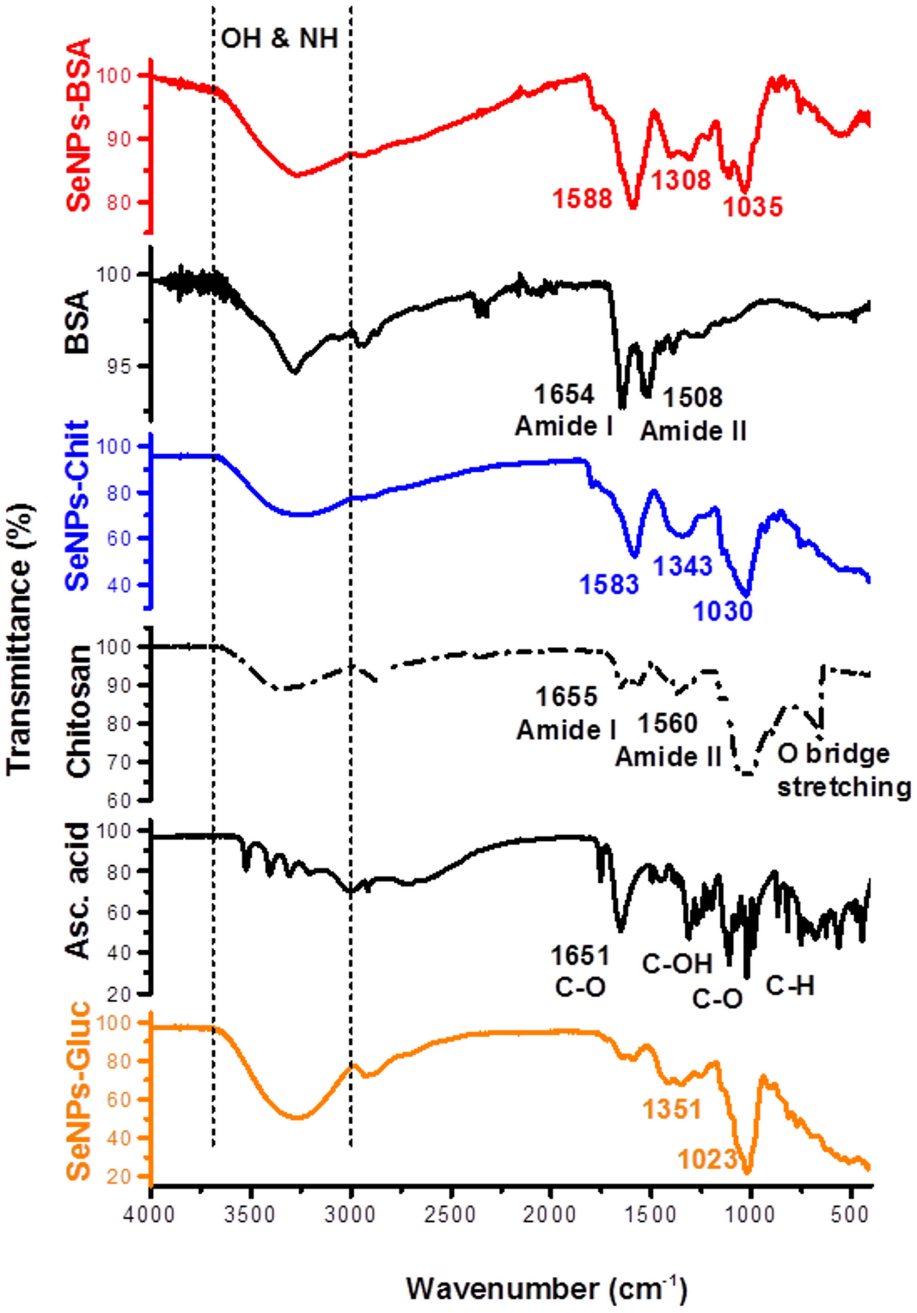
FTIR spectra of different SeNPs formulations and commercial compounds used in the synthesis procedures.

As it was expected for lyophilized samples, the dominant absorption of the IR incident beam from the O-H bond is recorded around 3,300 cm^−1^ (Figure 1). Besides this, several peaks can be noticed in all spectra: vibrations of C-O and C-C bonds around 1,050 cm^−1^, C-H bending vibrations around 1,350 cm^−1^, and peak at ~1,580 cm^−1^ that is much more pronounced in spectra of SeNPs-BSA and SeNPs-Chit. The first two peaks originate from functional groups dominant in the structure of ascorbic acid, glucose, and chitosan (Ibrahim et al., 2006; Bunaciu et al., 2009; Kumirska et al., 2010). If we considered the third peak from the aspect of composition, there is no clear analogy of its origin. Both BSA and chitosan belong to complex macromolecules which among others, exhibit characteristic amide I band at ~1,650 cm^−1^, amide II band at ~1,550 cm^−1^, and amide III band at ~1,250 cm^−1^ (Grdadolnik and Maréchal, 2001; Kumirska et al., 2010). Additionally, ascorbic acid displays a strong band around 1,650 cm^−1^ due to the C-O stretching vibrations. Nevertheless, in spectra of SeNPs obtained with these two stabilizers, only one peak appears at 1,580 cm^−1^. Since amide I and II bands originate from vibrations of C=O and N-H bonds and reflect the secondary structure of mentioned macromolecules, it is possible that during the synthesis procedure those structures are changed as a consequence of the adsorption on the surface of SeNPs, preventing them from agglomeration in a larger scale.

### XRD Characterization

**Figure 2:**
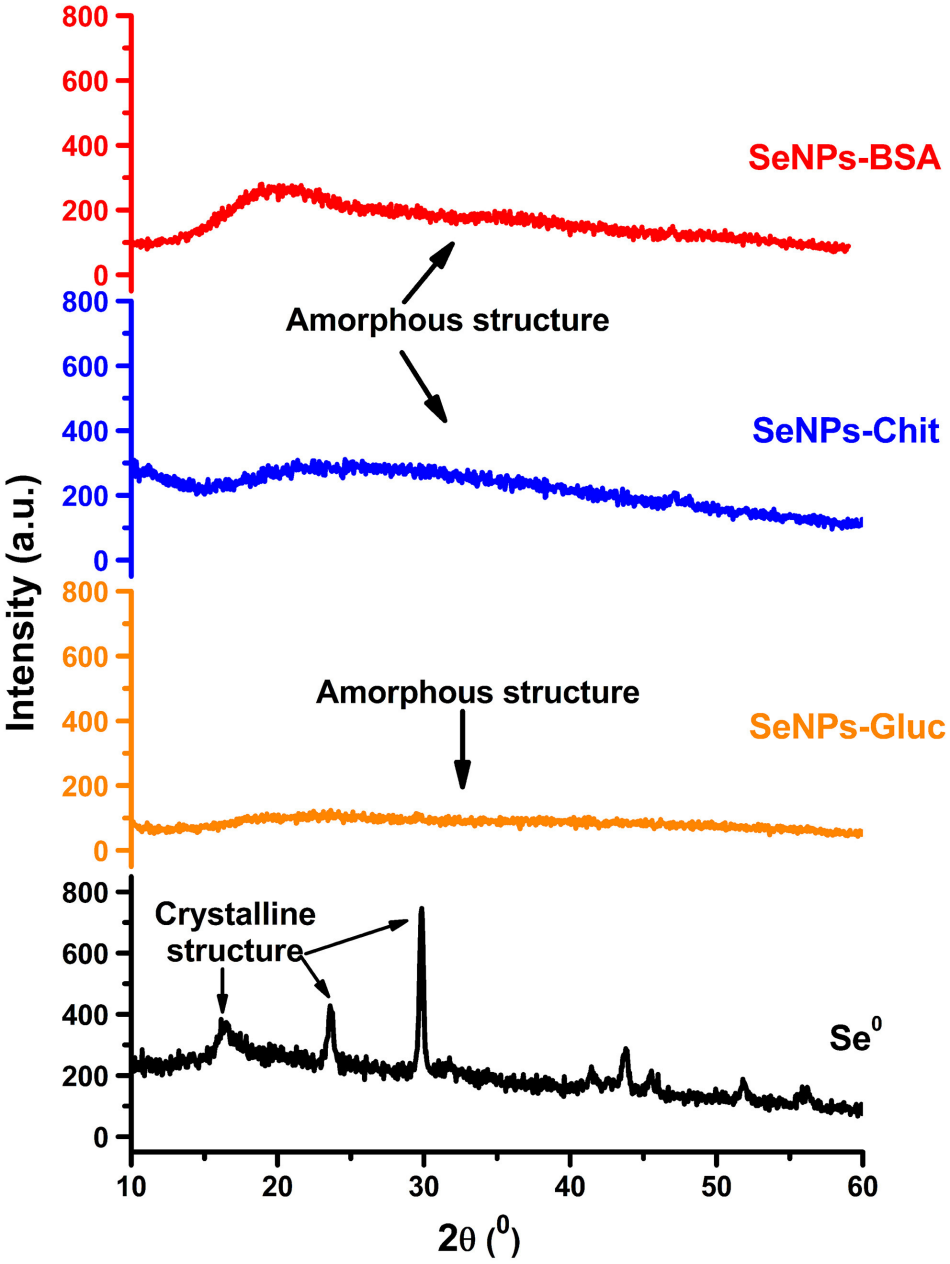
XRD patterns of different SeNPs formulations.

To evaluate the crystalline nature of obtained SeNPs, lyophilized samples were characterized by the XRD technique. The obtained diffractograms are given in Figure 2. Based on these diffractograms it can be seen that all samples exhibit short-range arrangement i.e., amorphous structure. In amorphous materials incident X-rays are scattered in many directions leading to a constant oscillation of signal line distributed in a whole range of 2θ instead of high intensity narrower peaks. These diffraction peaks as proof of the crystalline phase were not recorded. For comparison reason, the diffractogram of elemental Se is given in Figure 2. This sample was prepared by the same procedure as SeNPs-BSA and SeNPs-Chit but without employing any stabilizing agents. Several narrow peaks noticed for this sample point to its crystalline nature. All the peaks could be indexed according to the trigonal phase of elemental selenium (Shah et al., 2010). Since the trigonal form of Se is thermodynamically most stable, obtaining amorphous SeNPs can be challenging in procedures that involve elevated temperature. For example, some authors reported obtaining SeNPs with the crystalline structure in similar conditions such were used in the SeNPs-Gluc synthesis (Ingole et al., 2010; Koo et al., 2017). In addition, Zhang and others reported that heating of colloidal solution of amorphous SeNPs at 90°C caused the transformation to trigonal form (Zhang et al., 2012). Pinto et al. even shown that this transformation is even possible as a spontaneous process at room temperature during the time (Pinto et al., 2012). The crystallinity of SeNPs was proven as an important factor for its bioavailability and bioactivity (Zhang et al., 2012). Thus, to perform an adequate comparison of antimicrobial activity between samples, all of them needed to be amorphous.

### Morphological Characterization

**Figure 3:**
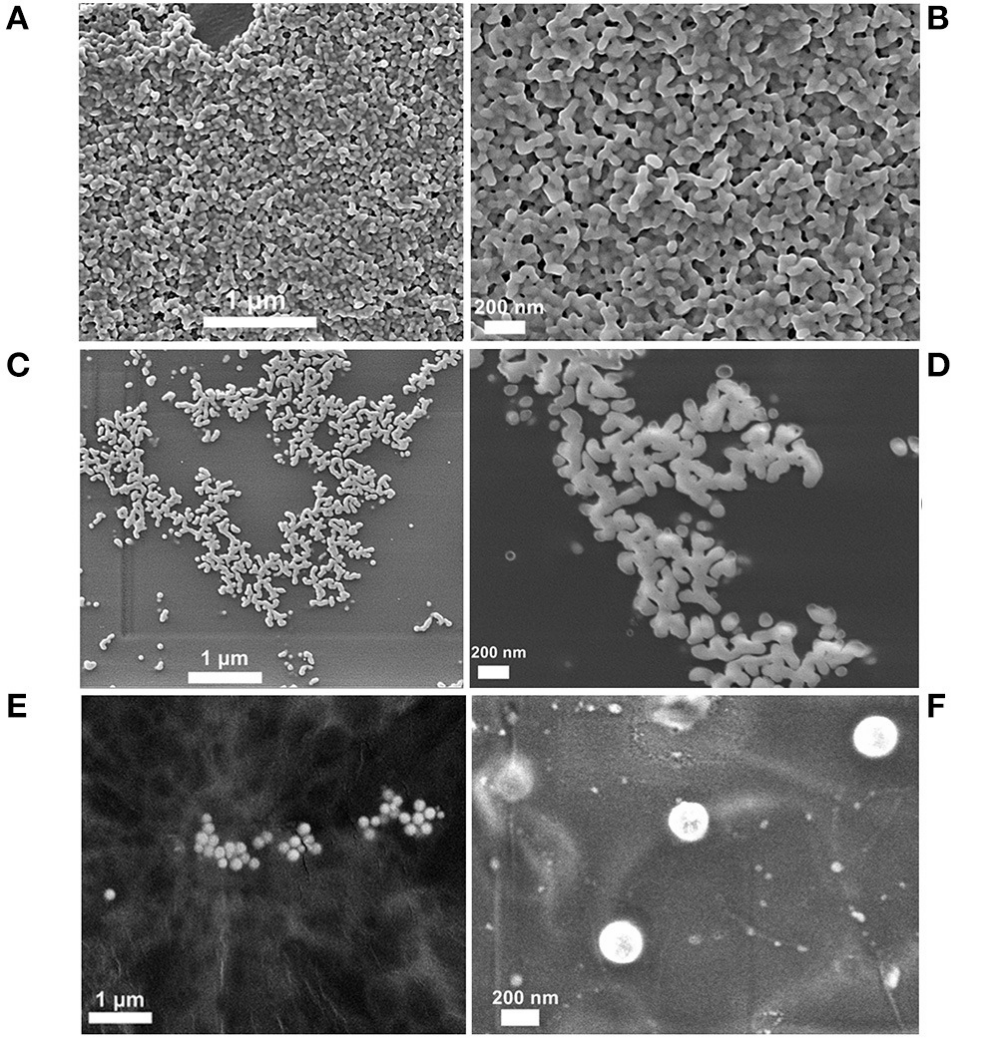
FE-SEM micrographs of different SeNPs formulations. **(A,B)** SeNPs-BSA, **(C,D)** SeNPs-Chit, **(E,F)** SeNPs-Gluc.

The morphology of nanoparticles is one of the three parameters that have the most significant influence on their interaction with biological entities. Results obtained from scanning electron microscopy of SeNPs are represented in Figure 3. The sample of SeNPs-Gluc possesses spherical morphology with a particle size of around 100–200 nm. When it comes to the other two samples, micrographs imply the agglomeration of particles at a higher level. This agglomeration could be attributed to the evaporation process, adhesive properties of stabilizers, degradation of the surface coating of the NPs since the stability of the chitosan is very often influenced by environmental conditions such as the lyophilization process. All these processes led to significant alterations of the morphology and properties of biomolecules-based coatings that further affect agglomeration. Nevertheless, the size of the individual particles can be estimated to be lower than in the case of SeNPs-Gluc, around 100 nm. In many previous works, the chemical reduction approach proved to be convenient for producing spherical and amorphous SeNPs (Hosnedlova et al., 2018; Sakr et al., 2018; Shi et al., 2020). Among other parameters (concentrations, the ratio between dispersion phases, mixing method, temperature, etc.), final morphology strongly depends on the choice of stabilizers. Spherical particles are most frequent since this morphology provides a lower level of surface energy. As it was stated earlier, one of the goals in this work was to compare SeNPs with the same morphology and with comparable size but with different surface chemistry.

### The Size Distribution and the Stability of Nanoparticle Suspensions

**Figure 4:**
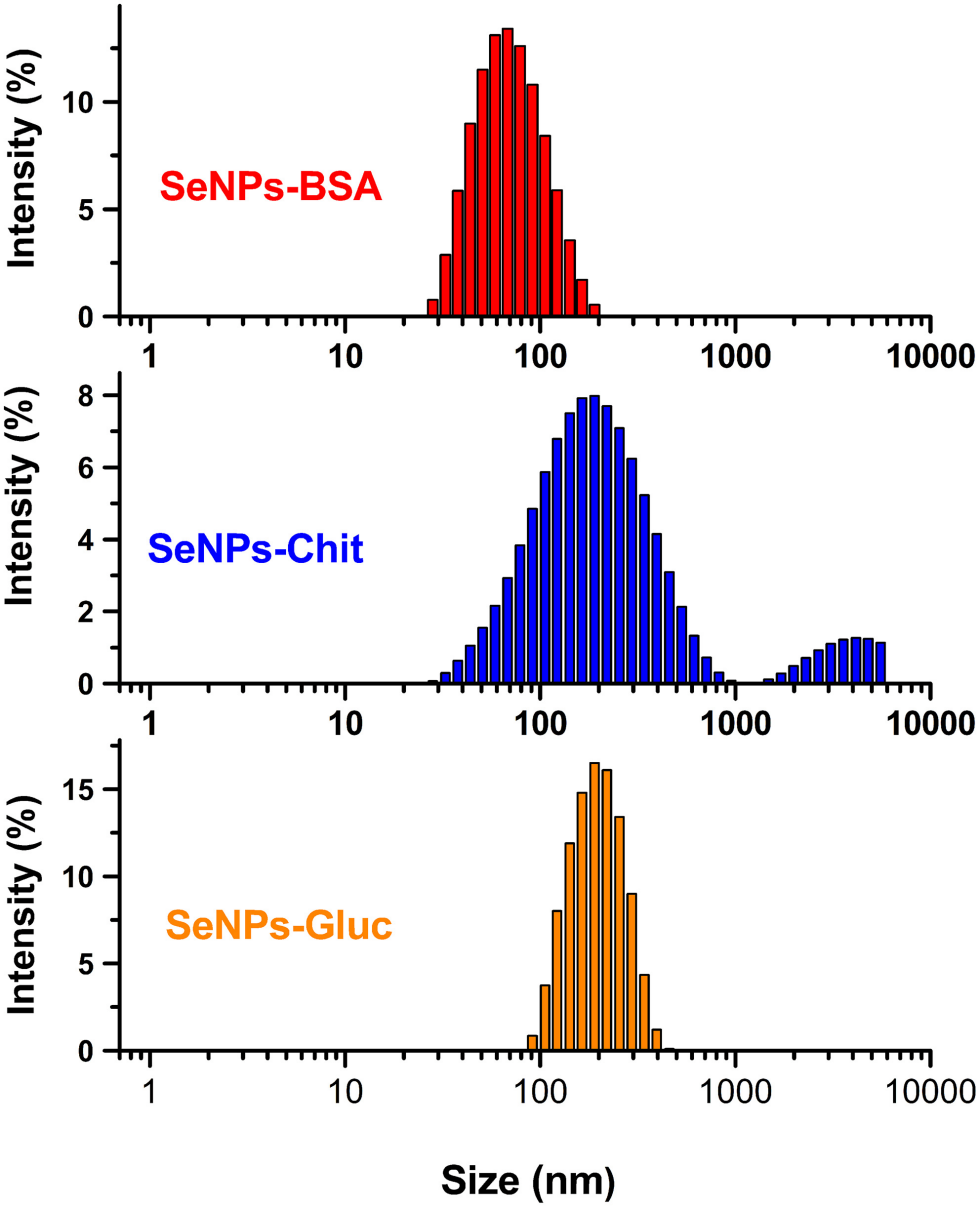
The size distribution of different SeNPs formulations, measured by diffracted light scattering.

DLS (Figure 4) and measurements of zeta potential (ZP) (Table 1) were conducted to gain insight into the parameters of SeNPs colloidal solutions. The ZP value is a very important parameter for the evaluation of the stability of nanoparticle suspensions (Honary and Zahir, 2013). When nanoparticles are stabilized with low molecular weight surfactants, mainly through electrostatic interactions, it is considered that ZP values higher than 25 mV point to very good stability (Shnoudeh et al., 2019). However, when stabilization is achieved with high molecular weight stabilizers (steric stabilization) ZP values of only 20 mV or lower can provide sufficient repulsive forces and good stabilization (Honary and Zahir, 2013). The values of zeta potential, obtained from three measurements for each SeNPs formulation, are given in Table 1.

**Table 1:** Values of zeta potential and average hydrodynamic radius for different SeNPs.

**Measurements\Formulations**	**SeNPs-BSA**	**SeNPs-Chit**	**SeNPs-Gluc**
Mean Zeta potential (mV)	+27 ± 3	+24 ± 1	−45 ± 1
Average hydrodynamic radius (nm)	76 ± 2	162 ± 3	199 ± 5

Based on the presented results it is expected that all samples possess good stability. Absolute values of zeta potential increases in order: SeNPs-Chit <SeNPs-BSA <SeNPs-Gluc While BSA and chitosan provide a positive charge of SeNPs surface, the formulation obtained by reduction with glucose exhibit a highly negative charge. These results confirmed that SeNPs are well covered/stabilized with both BSA and chitosan, providing free amino groups and net positive charge. The negative surface charge of particles prepared without stabilizing agents was also reported in two separate papers, for particles prepared with pulsed laser ablation (Guisbiers et al., 2016; Geoffrion et al., 2020). The highest value obtained for suspension of SeNPs-Gluc indicates that this suspension is the most stable. However, results obtained from particle size distribution and scanning electron microscopy revealed that some amount of the particles in this sample have a diameter above 200 nm, which increases the risk of particles sedimentation (Frens, 1972; Larsson et al., 2011). The size distribution of formulation prepared with chitosan indicates a bimodal distribution and the presence of a small fragment of significantly larger particles, over 1 micron. Considering the FESEM image of this sample, the formation of big agglomerates is most likely the reason for this wide distribution. The narrowest particle size distribution, as well as the smallest diameter (<100 nm), were observed in the formulation SeNPs-BSA. The values of the mean hydrodynamic radius obtained for all SeNPs formulations are listed in Table 1.

**Figure 5:**
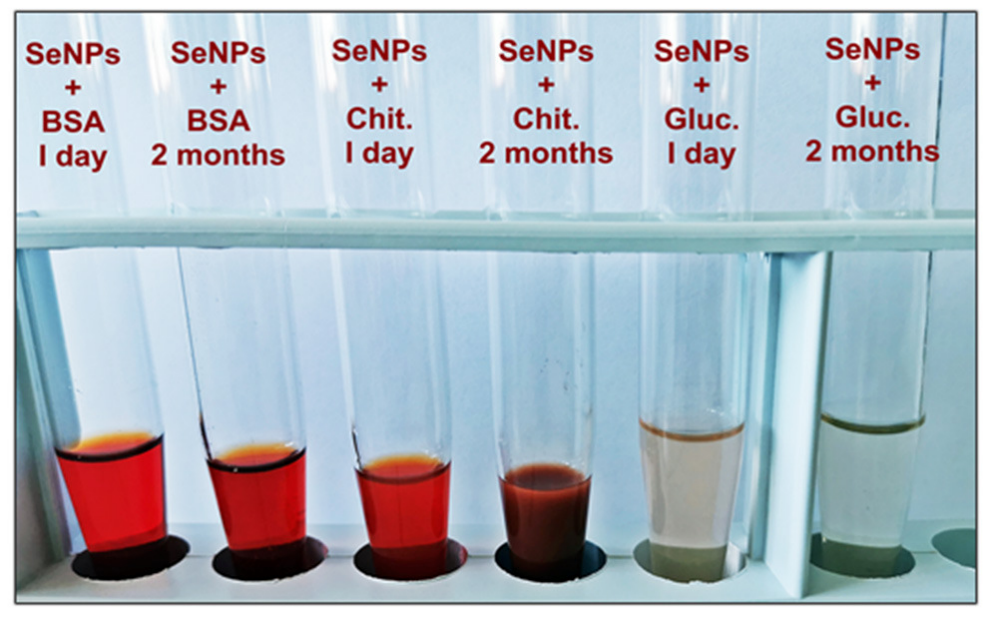
Macroscopic observation of SeNPs stability.

Further investigation of the stability of obtained colloidal solutions of SeNPs was conducted by visual observation for 2 months. The macroscopic observation of samples stability after 1 day and 2 months is given in Figure 5. As it can be seen, only sample SeNPs-BSA remained stable without the occurrence of turbidity or precipitation which is the case for SeNPs-Chit and SeNPs-Gluc, respectively. This result is in good agreement with those obtained from the DLS measurements. The stability of nanoparticle systems is important from the aspect of the application, particularly interaction with biological entities. The unstable systems are not reliable since their interaction can be easily changed upon particles agglomeration.

### Quantitative Analysis of Se in Obtained SeNPs Formulations

The quantitative determination of SeNPs in obtained colloidal solutions was performed by ICP-AES analysis and the following results were obtained: 624 μg/mL for SeNPs-BSA, 610 μg/mL for SeNPs-Chit, and 428 μg/mL for SeNPs-Gluc. These concentrations represent more than 90% of the theoretical yield. Considered from the composition of the nanoparticles and by the assumption that all stabilizing agents are well attached on the surface of Se, it could be estimated that concentrations of pure Se in SeNPs are 290 and 280 μg/mg for SeNPs-BSA and SeNPs-Chit, respectively. The confirmation of complete reduction of sodium selenite can be also found in the diffraction pattern of obtained lyophilized samples since none of the reflection, characteristic for this compound, was noticed (Youngren et al., 2013). Additionally, in FTIR spectra of all SeNPs formulations, the presence of a strong peak at 715 cm^−1^ was not detected, as in the case of commercial sodium selenite (Supplementary Figure 1). This intensive peak could be attributed to the vibrations of the Se-O bond.

### Antibacterial and Antifungal Activities of SeNPs

The ability of three different formulations of SeNPs to inhibit the growth of four Gram-positive and four Gram-negative common bacteria, as well as of yeast *C. albicans*, was investigated. As indicated in Table 2, SeNPs were most active against *C. albicans*, particularly, SeNPs-BSA and SeNPs-Chit yielded MICs as low as 25 μg/mL while SeNPs-Gluc induces the same effect at concentration 72 μg/mL (MIC concentrations presented in Table 2 refer to the concentrations of pure Se). In previous works, the activity of biogenic SeNPs, synthesized with *Bacillus sp*. Msh-1 (80–220 nm), was tested against *C. albicans*. Obtained MICs varied between 70 and 100 μg/mL and the authors described their results as good antifungal activity (Shakibaie et al., 2015b; Parsamehr et al., 2017). Furthermore, the SeNPs formulations presented in this work showed more potent activity against Gram-positive species. This behavior could be attributed to a significant difference in bacterial wall composition, including abundant pores and a thin layer of peptidoglycan. Many authors reported similar results so far (Guisbiers et al., 2015; Tran et al., 2016). However, in the paper presented by E. Cremonini et al. it was shown that biogenic selenium nanoparticles possess very good antimicrobial activity against clinical isolates of *P. aeruginosa* but lower efficacy toward *C. albicans* (Cremonini et al., 2016). Interestingly, in the same study, the authors compared the antimicrobial activity of biogenic and chemically synthesized SeNPs and obtained significantly better results for the first one. Since biosynthesized SeNPs have a special biogenic surface coating, the authors suggest that this is the reason for higher antimicrobial activity. Especially efficient were the chitosan-stabilized SeNPs, which is in agreement with observations that chitosan alone possesses potent activity against Gram-positive species (Goy et al., 2009). Recently, Rangrazi et al. tested the antimicrobial activity of chitosan-stabilized SeNPs with a diameter range of 50–105 nm (Rangrazi et al., 2020). They documented MICs of 137 and 274 μg/mL against *S. aureus* and *E. faecalis*, respectively, but did not observe any activity against Gram-negative species. When it comes to Gram-negative bacteria, *K. pneumoniae* was most susceptible to tested samples, whereas the lowest activity was exhibited against *S*. Abony and *P. aeruginosa*. Overall observed, formulation SeNPs-Gluc achieved the lowest activity against all microbial strains except for *K. rhizophila* and *E. coli*. For these bacteria, SeNPs-Gluc have demonstrated activity at a lower concentration in comparison with SeNPs-BSA.

**Table 2:** MIC values (μg/mL) of different SeNPs formulations and sodium selenite against standard microbial strains.

**Microorganism type**	**Strain**	**SeNPs-BSA**	**SeNPs-Chit**	**SeNPs-Gluc**	**Na_**2**_SeO_**3**_**
Gram-positive bacteria	*Staphylococcus aureus* ATCC 6538	100	100	290	100
	*Enterococcus faecalis* ATCC 29212	100	100	290	>400
	*Bacillus subtilis* ATCC 6633	200	100	290	>400
	*Kocuria rhizophila* ATCC 9341	200	100	145	200
Gram-negative bacteria	*Escherichia coli* ATCC 8739	400	200	290	>400
	*Salmonella* Abony NCTC 6017	400	400	>400	>400
	*Klebsiella pneumoniae* NCIMB 9111	200	200	290	>400
	*Pseudomonas aeruginosa* ATCC 9027	400	400	>400	>400
Fungi	*Candida albicans* ATCC 10231	**25**	**25**	**72**	400

*The bold values represent the most significant antimicrobial effect*.

All SeNPs formulations have expressed higher antimicrobial activity than sodium selenite against all tested strains (Table 2). This difference is most profound for *C. albicans, E. faecalis*, and *B. subtilis*. Based on this observation, it could be concluded that nanoparticle systems express activity through a different mechanism of action.

The use of nanoparticles as antimicrobial agents is a promising strategy, especially when dealing with chronic and nosocomial infections. The widespread usage of commercial antibiotics has led to the development of multidrug-resistant bacterial strains. Generally, various mechanisms of nanoparticles antimicrobial activity were recognized up to now: ROS generation, interaction with cell barrier (cell wall disruption and alteration in permeability), inhibition in the synthesis of proteins and DNA, expression of metabolic genes, etc. (Hemeg, 2017; Wang et al., 2017; Eleraky et al., 2020). When it comes to metal-based nanoparticles, the antimicrobial activity is very often attributed to ROS production (hydroxyl radicals, superoxide anions, and hydrogen peroxide). These ROS species can further inhibit DNA replication and amino acid synthesis as well as induce damage to the bacterial cell membrane (Hemeg, 2017). From the literature, the morphology of nanoparticles also plays the important role in its effectiveness against microbial species (Hong et al., 2016; Cheon et al., 2019). The main advantage of nanoparticles as antimicrobial agents could be described as their ability to simultaneously act through these multiple mechanisms. As a result, microbes are unable to develop resistance to these expressed mechanisms of action, contrary to commercial antibiotics.

Based on the literature data, a comparison of antimicrobial activity of differently stabilized SeNPs, have not been investigated in the past. However, a similar study was done regarding the anticancer activity of SeNPs stabilized with different amino acids. In this work, authors reported an increase in activity for system “decorated” with positively charged amino acid-Lysine in comparison with the other two systems where neutral and anionic amino acids were used (Feng et al., 2014). It is also interesting to consider the antimicrobial activity of different SeNPs samples from the zeta potential aspect. In opposite to the other two samples, SeNPs-Gluc possesses a highly negative surface charge as is shown in Table 1. The overall antibacterial activity of this sample is significantly lower than for the other two samples. According to some authors, positively charged NPs have greater potential in inhibition of bacterial growth due to the better attachment on bacterial (Pan et al., 2013; Fang et al., 2015). Conversely, negatively charged particles need to overcome repulsive interaction to adhere to the bacteria's surface. Arakha et al. reported that high concentrations of negatively charged NPs could induce significant antibacterial activity due to the phenomenon known as molecular crowding (Arakha et al., 2015). Here reported negatively charged SeNPs, exhibit more pronounced activity toward Gram-positive bacteria, which could be described by the lack of negatively charged LPS molecules in their cell walls and consequently the absence of mentioned electrostatic repulsions. If we consider that the same behavior was also observed for positively charged formulations of SeNPs, it could be concluded that electrostatic interaction between SeNPs and bacterial cell barrier is not a crucial step that determined their antimicrobial activity. Negatively charged regions on the cell's wall of Gram-negative bacteria are not sufficient to ensure attachment of positively charged SeNPs. The penetration channels in the cell wall of Gram-negative bacteria are very small allowing the entrance to macromolecules only. Furthermore, from the aspect of particle size, sample SeNPs-BSA should be most active, since these particles possess a smaller diameter. It is a known fact that the internalization of nanoparticles increases with the decrease in their size. In the work of Huang and coauthors, it has been reported that SeNPs with a mean diameter of 80 nm exhibit the best antimicrobial activity against methicillin-sensitive and methicillin-resistant *S. aureus* (Huang et al., 2019). The investigated diameter range in this study was from 40 to 200 nm, and polyvinyl alcohol (PVA) was used as a stabilizer. In contrast to this finding, the best overall antimicrobial activity was recorded for sample SeNPs-Chit, which possesses the wider distribution and significantly larger diameter than SeNPs-BSA. In the end, formulations SeNPs-Chit and SeNPs-Gluc have more comparable size but express remarkable different antimicrobial activity. Based on the results that are presented here it can be concluded that surface chemistry is the most influenced parameter that determines the antibacterial activity of SeNPs. Subsequent to surface chemistry is particle size, where further investigation must be done to precisely determine the thresholds for some mechanisms of antibacterial activity.

### Morphology Observation by Optical Microscopy

**Figure 6:**
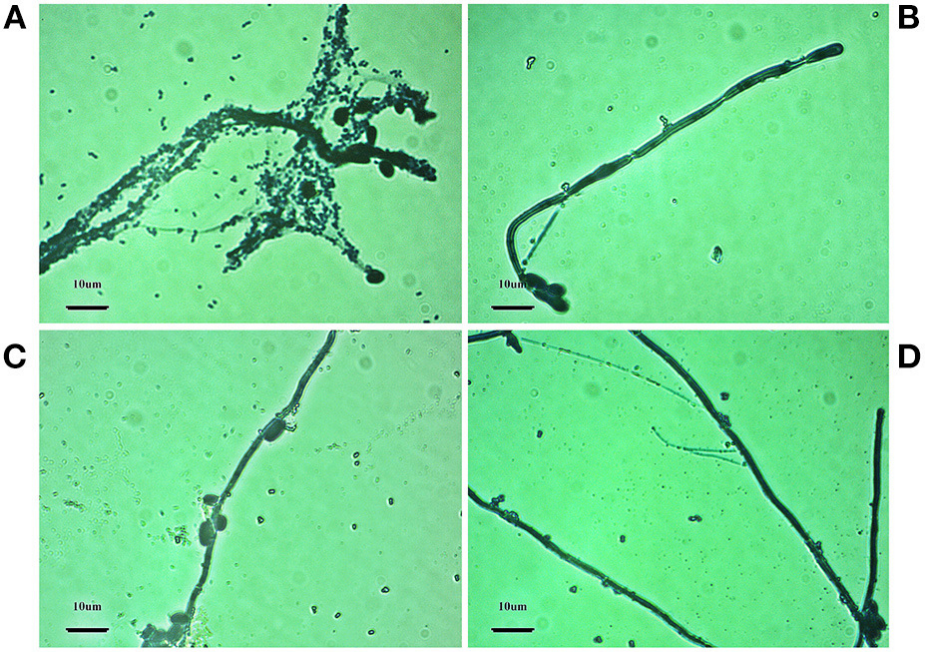
Light microscopy images showing the impact of SeNPs formulations on *S. aureus* and *C. albicans*: **(A)** control- prepared without the addition of any SeNPs formulation, **(B)** SeNPs-BSA, **(C)** SeNPs-Chit, **(D)** SeNPs-Gluc. Magnification bars 10 μm.

The impact on the morphology and growth of the pathogens *S. aureus* and *C. albicans*, after the treatment with SeNPs, has been done by OPTICA B-500MET light microscope. It is known that *C.albicans* can act synergistically with bacteria, especially with *S. aureus*, which has a strong affinity for *C. albicans* hyphae (Peters et al., 2010; Lin et al., 2013). It has been suggested by Kean et al. that *S. aureus* can form mycofilms, which is a new biofilm on already formed biofilm, using *C.albicans* hyphae as scaffolds (Kean et al., 2017). This interaction can greatly enhance virulence and resistance to antimicrobials of both pathogens (Kean et al., 2017; Carolus et al., 2019). Our results obtained by optical microscopy imply a significantly decreased number of *S. aureus* cell clusters attached to *C. albicans* hyphae after the treatment with all SeNPs formulations (Figure 6).

### Cytotoxicity of SeNPs

**Figure 7:**
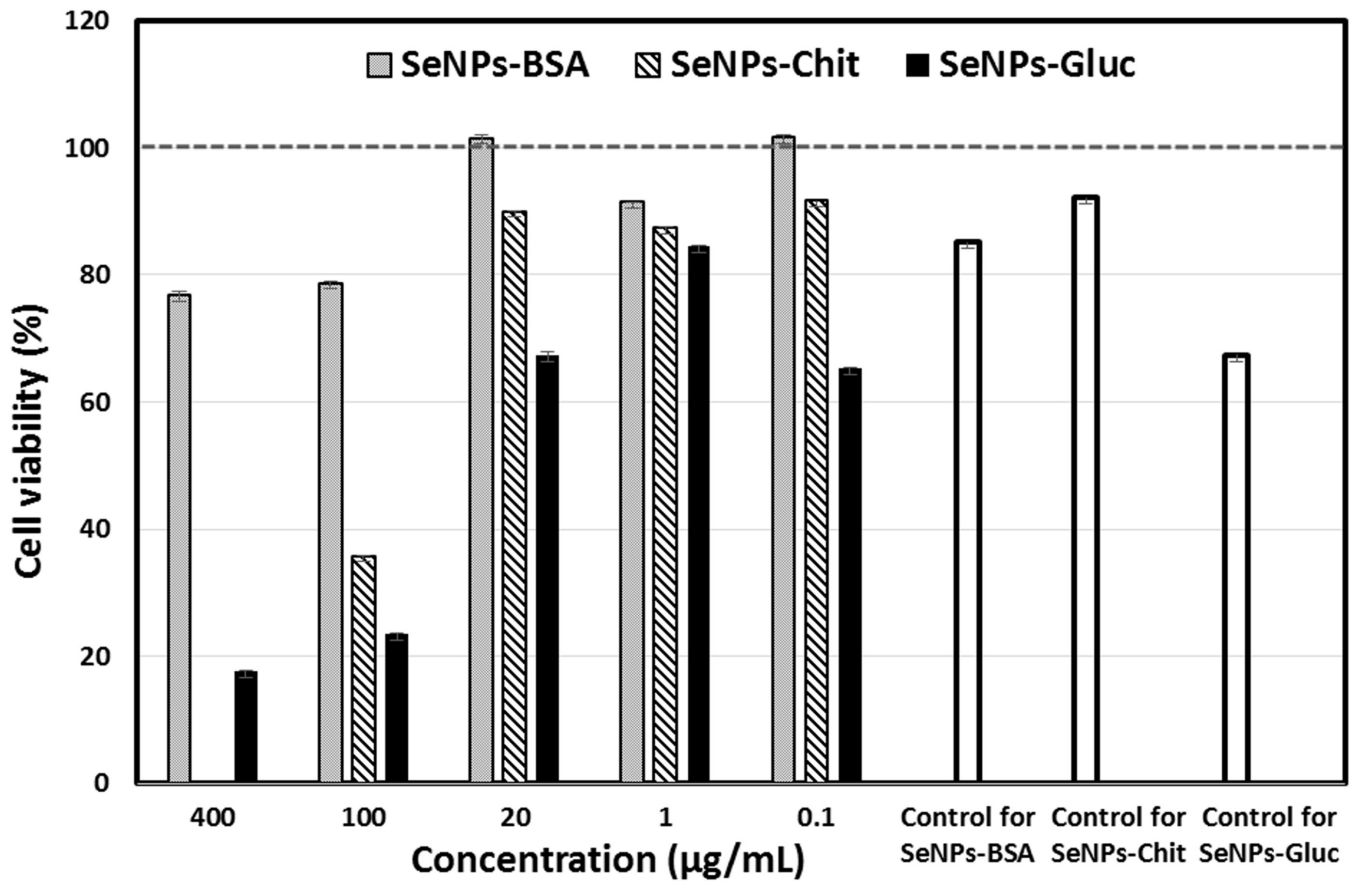
MRC-5 cell viability after the treatment with different formulations of SeNPs. Results were presented as relative to the untreated cells. Controls on the right side were prepared with appropriate amounts of BSA, chitosan, and glucose, as it was used in the synthesis of SeNPs.

To closely confirm the application potential of SeNPs, their effects on eukaryotic cells were investigated by MTT assay. The obtained results are presented in Figure 7 (presented concentrations refer to the concentrations of pure Se). A surprisingly large difference in cell viability was observed after their treatment with different formulations of SeNPs. SeNPs-BSA showed minor cytotoxicity at the highest concentrations (400 and 100 μg/ml). Lower doses (20, 10, 1 and 0.1 μg/ml) did not show cytotoxic effect (cells survival rate was above 80%). The dose of 100 μg/ml of SeNPs-Chit was cytotoxic, but at all lower concentrations, more than 80% of cells were viable. At 400 μg/ml, red-colored SeNPs could not be fully washed from the cells, therefore absorbance of samples treated with this concentration was not considered reliable. This effect was not present at lower concentrations, indicating possible excessive cellular uptake at high exposure of cells to SeNPs-Chit. SeNPs-Gluc has expressed more profound cytotoxic effects at almost all tested concentrations, except 1 μg/ml. The partial contribution of this phenomenon can be attributed to the fact that pure glucose solution, used for the synthesis of SeNPs-Gluc showed significant cytotoxicity compared to other controls. The other possible explanation could be found in the particle size since both SeNPs formulations with higher cytotoxicity rates possess larger particles (Figure 7).

Considering results obtained from antimicrobial and cytotoxicity testing as well as those regarding stability and size distribution, further investigation of antibiofilm activity has proceeded with formulation SeNPs-BSA.

### Monomicrobial and Dual-Species Biofilm Production Is Profoundly Inhibited by SeNPs

**Figure 8:**
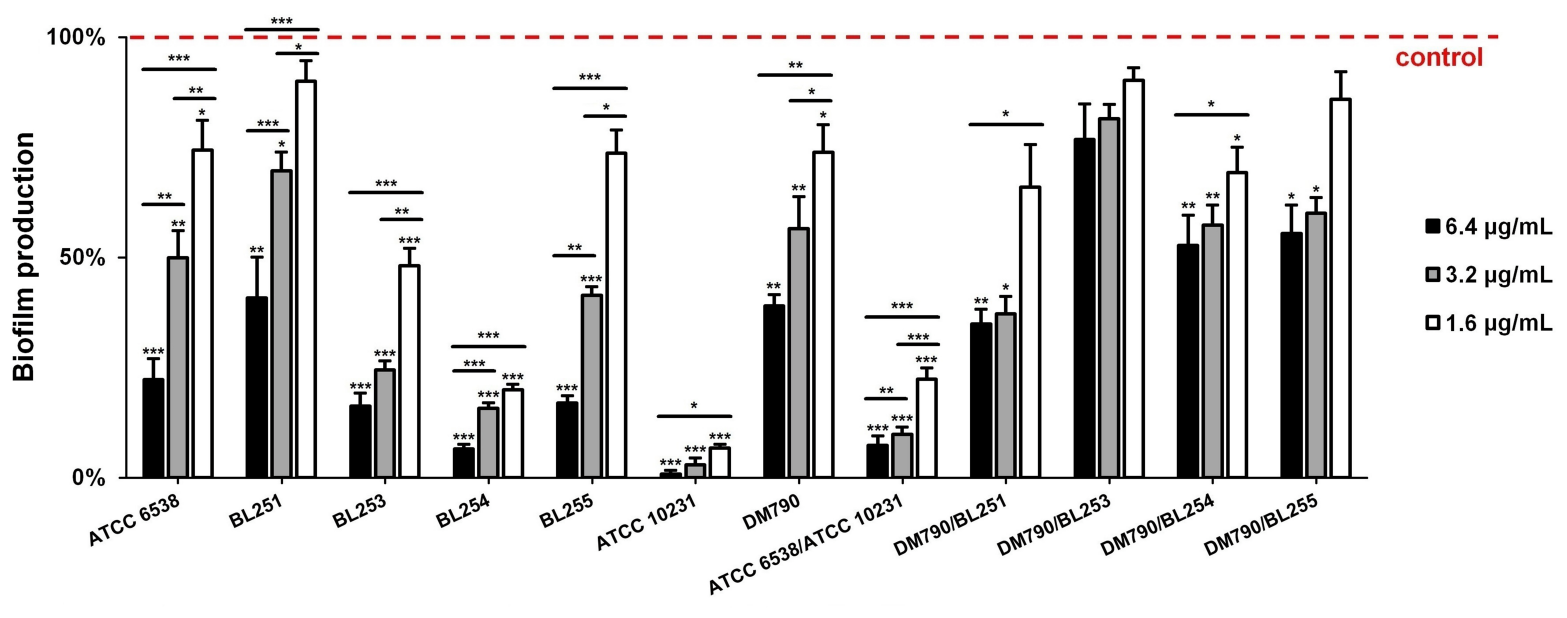
The activity of SeNPs-BSA against monomicrobial and dual-species biofilm production represented as the percentage of biofilm production compared to untreated control. *P < 0.05, **P < 0.01, ***P < 0.001.

A total of five strains of *S. aureus* and two strains of *C. albicans* were assessed on monomicrobial biofilm production, following the treatment with SeNPs-BSA. The significant dose-dependent inhibitory activity (*P* < 0.05; *P* < 0.01; *P* < 0.001) of SeNPs-BSA was observed in all tested strains (Figure 8). However, different strains responded variously. While the greatest level of suppression was noticed in *C. albicans* ATCC 10231 (93–99%) and *S. aureus* BL254 (80–93%), the biofilm production was the least affected in *S. aureus* BL251 (10–59%) and *C. albicans* DM790 (26–61%). In overall, the concentration of 6.4 μg/mL provided significant inhibition (*P* < 0.01; *P* < 0.001) in all tested strains, reducing the production by more than 50% (59–99%). These results are in agreement with some previous findings. For instance, in a study done by Shakibaie et al. biogenic SeNPs (80–220 nm) were tested against several clinical isolates of *S. aureus* and achieved a substantial level of inhibition at 2 μg/mL (41%) and 4 μg/mL (58%) (Shakibaie et al., 2015a). However, increasing the concentration to 16 μg/mL did not lead to any significant changes. Guisbers et al. have tested SeNPs synthesized by pulsed laser ablation in liquids (50–400 nm) against the biofilm production of *C. albicans* and observed dose-dependent inhibition up to 50% when increasing the concentration to 27 ppm (Guisbiers et al., 2017). In addition, they noticed that the smallest and crystalline SeNPs are the most active ones.

The biofilm structure provides various additional resistance and tolerance mechanisms to microorganisms, therefore, affecting its production could enhance the activity of antimicrobials and the host immune system, and subsequently stimulate the eradication of pathogens such as *S. aureus* and *C. albicans* (Koo et al., 2017). Most of the research articles deal with monomicrobial biofilm production, however, having in mind the clinical significance of *S. aureus* and *C. albicans* dual-species biofilm, and the additional tolerance and resistance mechanisms that this entity is equipped with, we decided to assess the activity of SeNPs-BSA against this type of biofilm, as well. The results revealed severe suppression of *S. aureus* ATCC 6538/*C. albicans* ATCC 10231 dual-species biofilm production (78–93%; *P* < 0.001), with significant dose-dependent effect (*P* < 0.01; *P* < 0.001) (Figure 8). Interestingly, the dual-species biofilm production was less affected by tested nanoparticles in communities of *S. aureus* and *C. albicans* DM790. Perhaps this clinical strain of *C. albicans* provides better protection to *S. aureus*, in comparison to *C. albicans* ATCC 10231, producing a denser biofilm matrix with more β-1,3-glucans, allowing the protected bacteria itself to produce more biofilm. At the same time, this could explain why the biofilm production of *S. aureus* was more inhibited when incubated without *C. albicans*.

**Figure 9:**
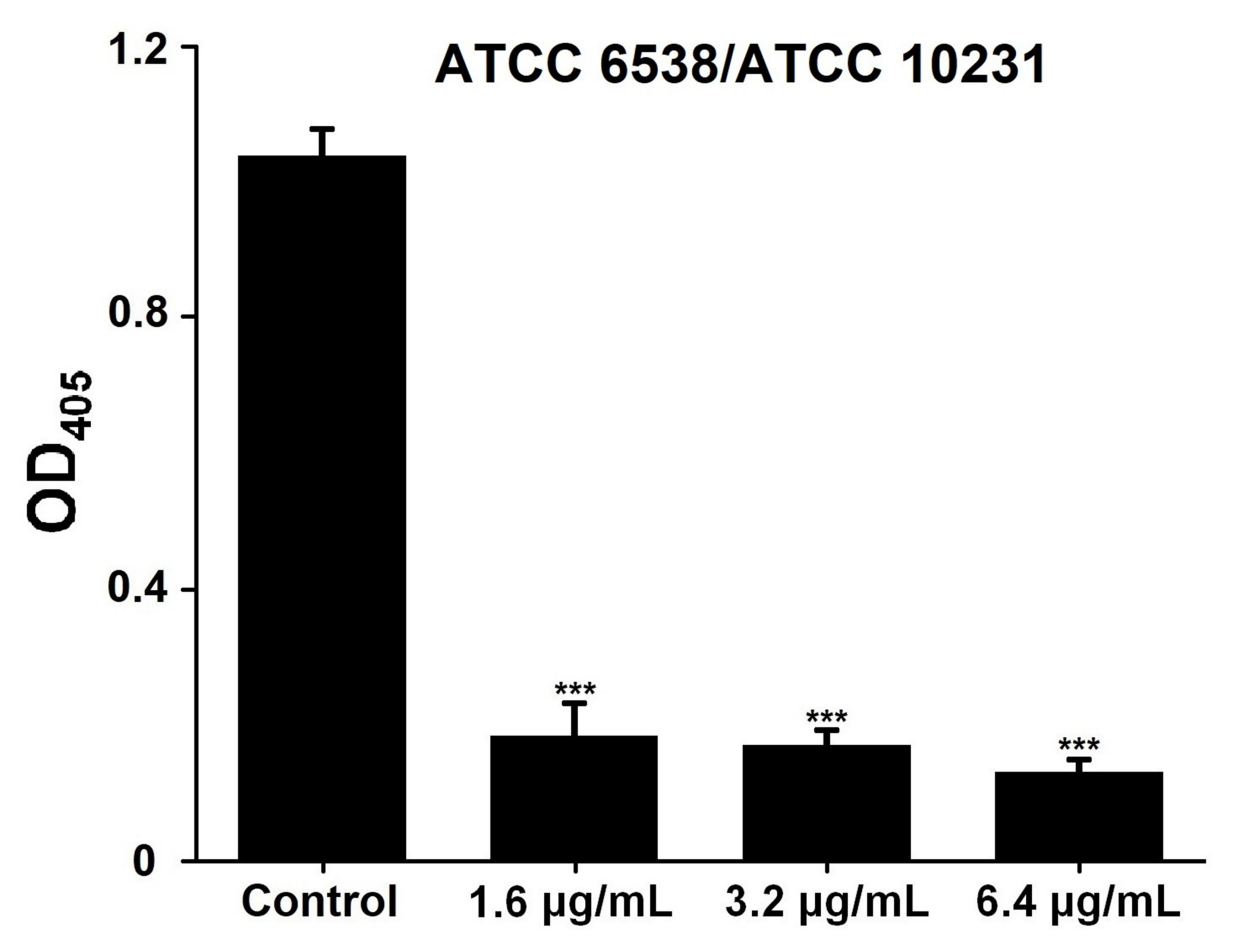
Biofilm cell viability of *S. aureus* ATCC 6538/*C. albicans* ATCC 10231 dual-species community after treatment with SeNPs-BSA. ***P < 0.001.

It should be noted that the antibiofilm activity of SeNPs-BSA, presented in this study (Figure 8), was demonstrated by using safranin that stains all biomass of the biofilm, including live and dead cells, and the extracellular polymeric substances (EPS), which may be overproduced by biofilm cells under the stress conditions (Bay et al., 2017). For this reason, we conducted an additional cell viability test (Figure 9), using TTC to measure exclusively live biofilm cells, both culturable and viable but non-culturable (VBNC) (Azeredo et al., 2017). Results obtained from this test, conducted on *S. aureus* ATCC 6538/*C. albicans* ATCC 10231 dual-species biofilm, revealed that the quantity of live cells is significantly inhibited, as is the whole biomass, ranging from 5.65- to 7.88-fold (*P* < 0.001), in a dose-dependent mode.

**Figure 10:**
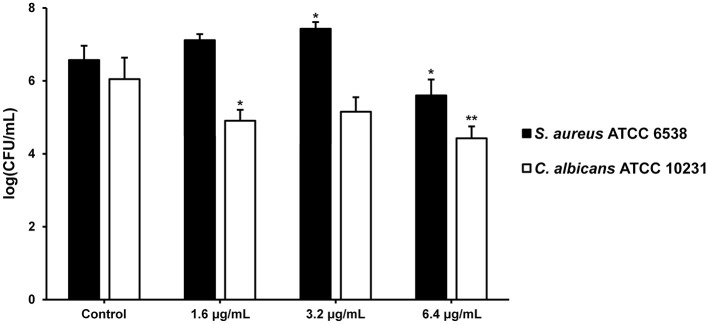
Relative numbers of *S. aureus* and *C. albicans* in mixed biofilms grown in the presence of SeNPs-BSA represented as logarithms of colony-forming units per milliliter [log(CFU/mL)]. *P < 0.05, **P < 0.01.

When it comes to the relative numbers of each species in this biofilm community, interestingly only the quantity of *C. albicans* viable culturable biofilm cells was reduced by SeNPs-BSA at all tested concentrations, most significantly at 6.4 μg/mL (*P* < 0.01) (Figure 10). The number of viable culturable biofilm cells of *S. aureus* was decreased only when the biofilm was grown in the presence of SeNPs-BSA at 6.4 μg/mL (*P* < 0.05). The reduction of viable cells was not as nearly severe as obtained when using TTC cell viability assay. However, it should be noted that TTC measures both culturable and non-culturable (VBNC–viable but non-culturable) biofilm cells, so it could be assumed that VBNC cells were more affected. This observation further contributes to the significance of obtained results, considering that VBNC cells provide the biofilms with an additional tolerance to high doses of antibiotics and other extreme conditions (Ayrapetyan et al., 2018).

The antimicrobial activity of nanoparticles against polymicrobial biofilm was reported only in a few papers so far. Su et al. recently reported inhibiting activity of chitosan nanoparticles loaded with coumarin against *S. aureus* and *C. albicans* monomicrobial and dual-species biofilm (Koo et al., 2017). The authors found that the effective concentration range for this system was 100–200 μg/mL. In another work done by H. Lara and J. Lopez-Ribot, AgNPs were able to inhibit the growth of the same dual-species biofilm at a concentration of 2 ppm (Lara and Lopez-Ribot, 2020). Despite the considerable interest in the use of AgNPs as an antimicrobial agent, there are some limitations for the safe application of this material such as cytotoxic effects and possible diminished therapeutic effects in prolonged treatment (Kostenko et al., 2010; Gliga et al., 2014; Kim, 2016).

**Figure 11:**
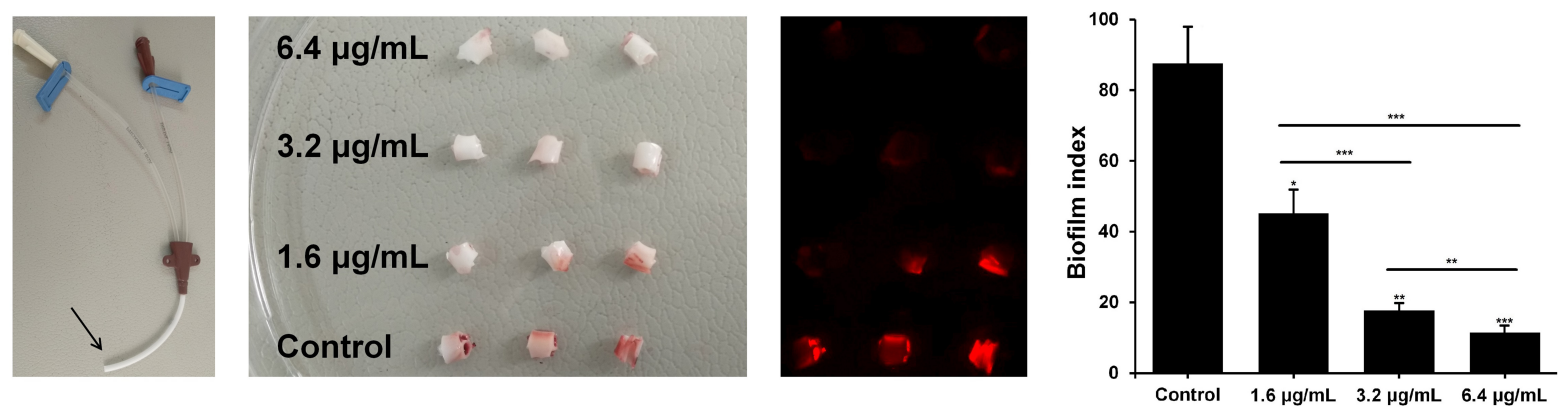
*S. aureus* ATCC 6538/*C. albicans* ATCC 10231 dual-species biofilm production on two-lumen Central Venous Catheter (CVC) in the presence of SeNPs-BSA. Coming from the left: digital image of CVC with a sampling region marked by arrow; digital image of tested CVC pieces; digital image used for color brightness quantification-red/green/blue (RGB) splitting, logarithmic transformation of R and G images, and brightness correction; quantified biofilm index with *P < 0.05, **P < 0.01, ***P < 0.001.

Further, biofilm infections are frequently associated with the use of indwelling medical devices. These types of infections are particularly dangerous, difficult to treat, and in most cases require complete removal of colonized devices or surgical excision of the infected tissue (Xu et al., 2017). Catheter-related bloodstream infections (CRBSIs) are common causes of severe hospital-acquired infections. Yearly mortality rates have been estimated at 7.7–23.1%. Besides, CVC is associated with considerably higher risk compared to the peripheral venous catheter (Saliba et al., 2018). Polymicrobial infections account for 9% of CRBSIs, with *S. aureus* and *C. albicans* being two of the most frequently isolated bacterial and fungal pathogens, respectively (Harriott et al., 2016; Thorarinsdottir et al., 2020). Therefore, we assessed the production of *S. aureus* ATCC 6538/*C. albicans* ATCC 10231 dual-species biofilms on radiopaque polyurethane vein-indwelling surface of CVC in the presence of SeNPs, as well. As can be seen from Figure 11, the experiment was performed in triplicate and the pieces, which represented positive control (at the bottom), were most abundant in reddish-colored patches, each representing individual biofilm structure. Significant dose-dependent reduction of biofilm production (*P* < 0.05; *P* < 0.01; *P* < 0.001) was revealed by using color brightness quantification (Figure 11). According to this, the dual-species biofilm was inhibited by 87 and 48%, when using SeNPs-BSA at 6.4 and 1.6 μg/mL, respectively.

Bearing in mind results obtained for antibiofilm activity, SeNPs-BSA can be considered as a promising candidate for inhibition of biofilm production, both monomicrobial and dual-species biofilm. This inhibition was observed in biofilms derived from clinical isolates of *S. aureus* and *C. albicans* and it was equally pronounced in real conditions i.e., surface of CVC. Further investigation should be done to determine the precise mechanism of biofilms inhibition by SeNPs and to examine the effects of different substrates on SeNPs antibiofilm activity.

## Conclusions

The antimicrobial activity of nanoparticles is a growing research field that demands a very complex evaluation strategy. When it comes to SeNPs diverse results have been reported so far. In this work, we have successfully synthesized three formulations of SeNPs using a simple, chemical reduction approach. Particles were obtained in the form of colloidal solutions and within the size range of 75–200 nm. The influence of surface chemistry on antimicrobial activity was investigated on eight standard bacterial strains and one yeast strain (*S. aureus, E. faecalis, B. subtilis, K. rhizophila, E. coli, S*. Abony, *K. pneumoniae, and P. aeruginosa, C. albicans*). The *C. albicans* was most sensitive to SeNPs, with detected MIC values of 25 μg/mL for SeNPs-BSA and SeNPs-Chit, and 72 μg/mL for SeNPs-Gluc. When it comes to bacterial strains, it was noticed higher activity against Gram-positive bacteria for all formulations of SeNPs. Also, it was shown that the positive surface charge of SeNPs provides the better antimicrobial activity. Finally, the synergistic effect of chitosan was more pronounced than the effect of particle size. All formulations of SeNPs expressed higher antimicrobial activity, particularly against *C. albicans*. In addition, cytotoxicity of SeNPs toward MRC-5 cells was examined and a significant difference was noticed. Survival of cells after the treatment with SeNPs increased in the following order: SeNPs-Gluc <SeNPs-Chit <SeNPs-BSA. Based on the results obtained for stability, particle size distribution, and cytotoxicity SeNPs stabilized with BSA were further used for investigations of antibiofilm activity. This investigation was done on several monomicrobial and dual-species biofilms, grown from clinical isolates of *S. aureus* and *C. albicans*. SeNPs-BSA displayed significant inhibition on all biofilms in a dose-dependent manner in comparison with control samples. The most expressed activity was detected for biofilms grown from *S. aureus* ATCC 6538 and *C. albicans* ATCC 10231, where inhibition of more than 90% was recorded in a concentration range as low as 1.6–6.4 μg/mL. The mentioned bacteria strain were additionally used for testing the inhibition of dual-species biofilm formation on polystyrene surface of 96-well microtiter plates and radiopaque polyurethane Central Venous Catheter (CVC). Similar to monomicrobial biofilm, here it was also detected inhibition of 80% within the same concentration range. Findings presented in this study will contribute to a better understanding in the field of SeNPs design and evaluation, particularly the influence of surface chemistry on the antimicrobial activity as well as in the activity of SeNPs against dual-species biofilm grown from clinical isolates.

## Data Availability Statement

The original contributions presented in the study are included in the article/Supplementary Material, further inquiries can be directed to the corresponding author/s.

## Author Contributions

NF: conceptualization, data curation, formal analysis, investigation, methodology, and writing–original draft preparation. DU: data curation, formal analysis, investigation, methodology, and writing–original draft preparation. MM: data curation, investigation, methodology, validation, writing–reviewing, and editing. KZ and LL: data curation, investigation, validation, writing–reviewing, and editing. AB: funding acquisition, project administration, resources, supervision, writing–reviewing, and editing. MS: conceptualization, investigation, methodology funding acquisition, project administration, resources, supervision, writing–reviewing, and editing. All authors: contributed to the article and approved the submitted version.

## Conflict of Interest

The authors declare that the research was conducted in the absence of any commercial or financial relationships that could be construed as a potential conflict of interest.
