# Supplementary material for: Comparative Study of the Antimicrobial Activity of Selenium Nanoparticles With Different Surface Chemistry and Structure
Source: Front Bioeng Biotechnol. 2021 Jan 25;8:624621. doi: 10.3389/fbioe.2020.624621 (PMC7869925; doi:10.3389/fbioe.2020.624621)
Supplement: Supplementary file 1 [file Table_1.DOCX]

Supplementary Material

**Supplementary Table 1.** Origin of clinical isolates

| **Species** | **Strain** | **Origin** | **Biological sample** |
| --- | --- | --- | --- |
| ***S. aureus* (MRSA)** | BL251 | Beo-Lab laboratory | nose swab |
| ***S. aureus* (MRSA)** | BL253 | Beo-Lab laboratory | ear swab |
| ***S. aureus* (MRSA)** | BL254 | Beo-Lab laboratory | nose swab |
| ***S. aureus* (MRSA)** | BL255 | Beo-Lab laboratory | nose swab |
| ***C. albicans*** | DM790 | Clinical Hospital Center  Dragiša Mišović | sputum |


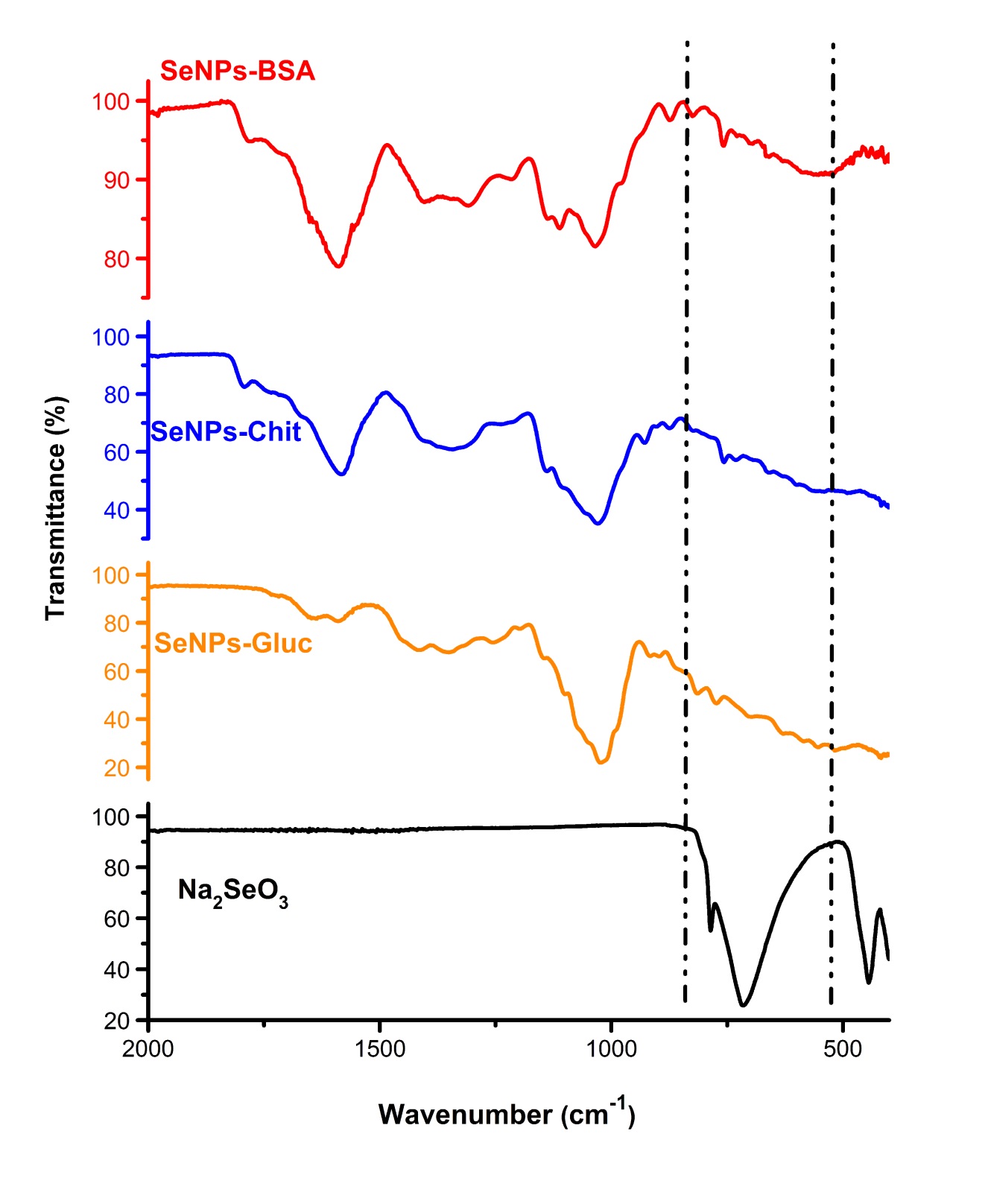


**Supplementary Figure 1.** The FTIR spectra of three different SeNPs formulation along with sodium selenite. Coming from the top: spectrum of SeNPs stabilized with bovine serum albumin (SeNPs-BSA); spectrum of SeNPs stabilized with chitosan (SeNPs-Chit); spectrum of SeNPs prepared by reduction with glucose (SeNPs-Gluc); and commercial sodium selenite (Na2SeO_3_) used as a precursor. For better comparison, spectra are given in region of wavenumbers were sodium selenite exhibit peaks.
